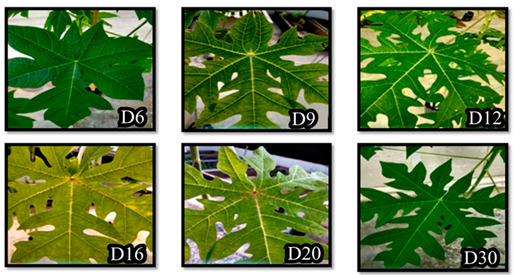# Characterisation and Mutagenesis Study of An Alternative Sigma Factor Gene (*hrpL*) from *Erwinia mallotivora* Reveal Its Central Role in Papaya Dieback Disease

**DOI:** 10.3390/biology9100323

**Published:** 2020-10-03

**Authors:** Amin-Asyraf Tamizi, Norliza Abu-Bakar, Aimera-Farhana Samsuddin, Lina Rozano, Rohaiza Ahmad-Redzuan, Abdul-Munir Abdul-Murad

**Affiliations:** 1 Agri-Omics and Bioinformatics Programme, Biotechnology and Nanotechnology Research Centre, Malaysian Agricultural Research and Development Institute Headquarters, Serdang 43400, Selangor, Malaysia; aminasyraf@mardi.gov.my (A.-A.T.); rozalina@mardi.gov.my (L.R.); rohaiza@mardi.gov.my (R.A.-R.); 2 School of Biosciences and Biotechnology, Faculty of Science and Technology, Universiti Kebangsaan Malaysia, Bangi 43600, Selangor, Malaysia; aimera.farhana@gmail.com (A.-F.S.); munir@ukm.edu.my (A.-M.A.-M.)

**Keywords:** bacterial disease, Malaysia, papaya, transcriptome sequencing

## Abstract

**Simple Summary:**

*Erwinia mallotivora* is the causal agent of papaya dieback disease in Malaysia, and its pathogenicity is less appreciated, especially from the molecular perspective. Our previous investigations proved that the *hrpL*/*rpoE* gene was one of the significant differentially expressed genes (DEGs) during early infection of *E. mallotivora* in papaya, suggesting this particular gene is important for infection. In this study, an in-depth analysis was performed using bioinformatics software on *hrpL* from *E. mallotivora* (*EmhrpL*) and its encoded protein (*Em*HrpL) obtaining crucial information including the conserved function and sequence motif, protein structural similarity with related homologs, and the possibility of being inhibited by a cognate inhibitor. Moreover, knockout (insertional mutational on DNA sequence) of the *hrpL* gene had caused mutant *E. mallotivora* (Δ*EmhrpL*) to be avirulent in four-month-old papaya plants. Here, the conclusion was that *Em*HrpL is indeed a necessary factor in *E. mallotivora* pathogenicity, and the findings on the potential inhibitor of this protein are useful for future studies to formulate a papaya dieback disease management programme.

**Abstract:**

The alternative sigma (σ) factor E, RpoE or HrpL, has been reported to be involved in stress- and pathogenicity-related transcription initiation in *Escherichia coli* and many other Gram-negative bacteria, including *Erwinia* spp. and *Pseudomonas* spp. A previous study identified the *hrpL*/*rpoE* transcript as one of the significant differentially expressed genes (DEGs) during early *E. mallotivora* infection in papaya and those data serve as the basis of the current project. Here, the full coding DNA sequence (CDS) of *hrpL* from *E. mallotivora* (*EmhrpL***)** was determined to be 549 bp long, and it encoded a 21.3 kDa HrpL protein that possessed two highly conserved sigma-70 (σ^70^) motifs—σR2 and σR4. Nucleotide sequence alignment revealed the *hrpL* from *E. mallotivora* shared high sequence similarity to *rpoE*/*hrpL* from *E. tracheiphila* (83%), *E. pyrifoliae* (81%), and *E. tasmaniensis* (80%). Phylogenetics analysis indicated *hrpL* from *E. mallotivora* to be monophyletic with *rpoE*s/*hrpL*s from *Pantoea vagans*, *E. herbicola*, and *E. tracheiphila*. Structural analysis postulated that the *E. mallotivora*’s alternative σ factor was non-transmembranic and was an extracytoplasmic function (ECF) protein—characteristics shared by other σ factors in different bacterial species. Notably, the protein–protein interaction (PPI) study through molecular docking suggested the σ factor could be possibly inhibited by an anti-σ. Finally, a knockout of *hrpL* in *E. mallotivora* (Δ*EmhrpL*) resulted in avirulence in four-month-old papaya plants. These findings have revealed that the *hrpL* is a necessary element in *E. mallotivora* pathogenicity and also predicted that the gene can be inhibited by an anti-σ.

## 1. Introduction

Alternative RNA polymerase sigma (σ) factors (Rpo) are essential small proteins required for translational initiation of other genes in certain pathways specifically related to stress tolerance, bacterial–host interaction, and pathogenicity [1,2,3,4]. Lambert et al. [5] proved that Rpo is important for specific binding of RNA polymerase to specific gene promoters and its crucial role in disease incidence has been discussed by Helmann [6]. Prior to the initiation of gene expression of a certain pathway, RNA polymerase recruits a sigma factor (along with a few other factors) and forms a holoenzyme before it can bind to the gene promoter region [6].

In several Gram-negative pathogens—as reported in the *Shigella*, *Salmonella*, *Erwinia*, and *Pseudomonas* genera—the type III secretion system (T3SS) is the major conserved infection mechanism employed during pathogenicity [7,8]. The T3SS is a complex system where a group of hypersensitive response and pathogenicity (*hrp*) genes work synchronously during a disease event. Naturally, this system is bound to RNA polymerase transcription initiation which involves the Rpo [6,7,9]. Once initiated, the T3SS transcribes various protein elements in the *hrp* family complex to assemble tiny needles known as harpins, which are used to contact host cells [10]. The secretion of effectors through T3SS is said to occur when various proteins, lytic enzymes, and ions are transferred across the cell membranes from the pathogen into the host cells, marking the start of an invasion [10,11,12].

The gene encoding an Rpo is also referred to as *hrpL* depending on the bacterial species [6,13]. The alternative σ factor plays a major role in regulating the T3SS gene by interacting with -10 and -35 motifs on the promoter region of the regulated genes [14]. It was demonstrated that the HrpL alternative σ factor activates HrpA, HrpN, and DspE effector genes in *D. dadantii* (formerly known as *Erwinia chrysanthemi*), which is a pathogen responsible for soft rot disease in several important crops [15,16].

*Erwinia mallotivora* has been identified as the pathogen that causes papaya dieback disease in Malaysia, responsible for the decline in national papaya export for almost two decades [17]. Nevertheless, knowledge of the virulence mechanism of *E. mallotivora* at the molecular level is relatively limited. In *E. mallotivora*, T3SS was first found from its draft genome sequence [18] and later reported in proteomics and transcriptomics studies [19,20]. Based on these, herein is a detailed description of a single gene associated with pathogenicity, *hrpL*, from *E. mallotivora,* which is thought to be the ‘master control’ of the T3SS. In this article, the gene *hrpL* from *E. mallotivora* is referred to as *EmhrpL* and its protein is referred to as *Em*HrpL.

## 2. Materials and Methods

### 2.1. Retrieval of EmhrpL Gene Sequence

The nucleotide information related to *rpoE*/*hrpL* of *E. mallotivora* was obtained from our previous RNA-seq experiment [20] and the full-length coding DNA sequence (CDS) of the *EmhrpL* gene was determined using the NCBI Open Reading Frame (ORF) finder (https://www.ncbi.nlm.nih.gov/orffinder/). The *hrpL* CDS of *E. mallotivora* was then deposited at GenBank (https://www.ncbi.nlm.nih.gov/genbank/) under accession number MK205448. The *E. mallotivora* draft genome data (GenBank accession no.: JFHN01000044) reported by Ahmad-Redzuan et al. [18] were also utilised to validate the sequence of *EmhrpL*.

### 2.2. Sequence Analysis, Gene Characterisation, and Phylogenetic Inferring of EmhrpL Gene

The CDS was searched against the database in the BLASTn program (https://blast.ncbi.nlm.nih.gov/Blast.cgi) and a total of 18 *hrpL*/*rpoE* sequences from different Gram-negative bacteria were obtained. These sequences were aligned using Clustal W (BioEdit, Raleigh, NC, USA) and the phylogenetic tree (Maximum Likelihood) was inferred using MEGA7 software (Pennsylvania State University, University Park, PA, USA) with the bootstrap value set to 1000. The percentage of trees in which the associated taxa clustered together is depicted next to the branches. The initial tree for the heuristic search was obtained automatically by applying Neighbor-Join and BioNJ algorithms to a matrix of pairwise distances estimated using the Maximum Composite Likelihood (MCL) approach and then, selecting the topology with superior log likelihood value.

The ExPASy translate tool (SIB Bioinformatics Resource Portal, Lausanne, Switzerland) was employed to translate *EmhrpL* nucleotide to its amino acids and protein homology analysis was done using the Protein Fold Recognition Server (Phyre2, London, UK). The Pfam database (http://pfam.xfam.org) and NPS@ server (https://npsa-prabi.ibcp.fr) were employed to study important protein motifs and domains on *Em*HrpL. The amino acid sequence was submitted to PSORTb version 3.0.2 (https://www.psort.org/psortb/) for the prediction of protein localisation.

### 2.3. Molecular Docking Analysis of EmHrpL with an Anti-σ Factor

Molecular docking of *Em*HrpL with anti-σ factor RseA of *Escherichia coli* was conducted using ZDOCK online server version 3.0.2 (http://zdock.umassmed.edu/). The structure of *Em*HrpL was predicted by SWISS-MODEL (https://swissmodel.expasy.org/) and deposited with repository number A0A014MCI7 using the crystal structure of RNA polymerase sigma-E factor (PDB ID: 1OR7) chain A as a template, with 25.31% sequence identity and 33% sequence similarity. The resulting *Em*HrpL model was docked with the anti-σ factor RseA, represented by chain C of the 1OR7 structure, including hydrogen bond interactions and salt bridge formations from 1OR7 as the contacting residues. Contact filtering had removed 1986 predictions out of 2000 from ZDOCK output files. *Em*HrpL was set as stationary and 1OR7_C was set to move.

### 2.4. Competent Cell Preparation, Construct Development, and Mutagenesis and Mutant Selection

**Figure 1:**
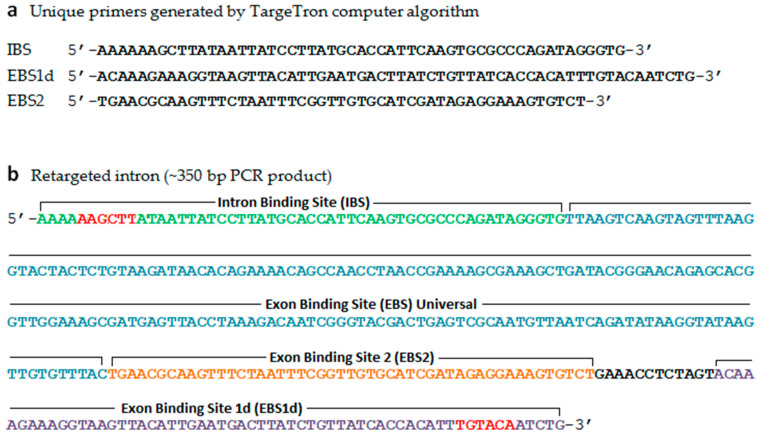
Sequences of Targetron^®^ unique primers and retargeted intron. (**a**) Unique primers were generated by TargeTron^®^ algorithm (www.sigmaaldrich.com/targetronaccess) and used to produce retargeted intron for gene reverse splicing; (**b**) Sequence of the retargeted intron (350 bp) containing *Hind*III (AAGCTT) and *Bsr*GI (TGTACA) restriction sites generated by the three unique primers through 1-step assembly PCR.

*Erwinia mallotivora* with mutated *hrpL* gene (Δ*EmhrpL*) was developed using the TargeTron^®^ Gene Knockout System (Sigma-Aldrich, St. Louis, MO, USA) according to the manufacturer’s protocol. The knockout system is a mutagenesis system based on the group II intron of the *ltrB* gene of *Lactobacillus lactis*. First, identification of the Lltr group II intron site within the *EmhrpL* ORF was carried out using an algorithm accessible from the Sigma-Aldrich TargeTron^®^ website (www.sigmaaldrich.com/targetronaccess), and sets of unique primers were exclusively generated by the program (Figure 1a). These primers were synthesised and utilised to generate a PCR fragment (350 bp) through overlap PCR reaction (1-step assembly PCR) that later was used in the development of a functional cassette for *hrpL*-targeted gene reverse splicing (Figure 1b). This generated a PCR fragment (retargeted intron) with *Hin*dIII and *BsrG*I restriction sites at the 5′ and 3′ UTR region, which was cloned into an intermediate plasmid. The plasmid was then digested with *Hin*dIII and *BsrG*I restriction enzymes and subcloned into a pACD4K-C linear vector to form a final construct (pΔ*hrpL*) that would be ultimately used for generating the Δ*EmhrpL* strain. Competent cells of the *E*. *coli* DH5α strain were transformed with the pΔ*hrpL* plasmid and selected (for propagation) on LB agar plates containing 25 μg/mL of chloramphenicol. Purification and retrieval of all plasmid DNAs were carried out using a NucleoBond plasmid extraction kit (Macherey-Nagel GmbH & Co., Doren, Germany).

Prior to transforming *E. mallotivora* with any plasmids, electrochemically competent cells were prepared. To produce competent *E. mallotivora* cells, the bacteria were cultured at 37 °C until attaining an optical density of 0.8 at 600 nm. Then, the cells were harvested by centrifugation at 12,000× *g* for 2 min and washed three times with 0.5 M sucrose and finally, suspended in 0.5 M sucrose. Plasmids of interest were transformed into the competent cells via electroporation using Bio-Rad micropulser (Bio-Rad) at 2.5 kV and transformed cells underwent selection on chocolate LB agar containing an appropriate antibiotic.

As the TargeTron^®^ system plasmid requires the use of a T7 promoter for targeting and mutation of selected genes in the bacterium of interest, *E. mallotivora* was first transformed with pAR1219, a pBR322-based vector, which expresses T7 RNA Polymerase under the control of the IPTG inducible lac UV5 promoter. At this first step, the *E. mallotivora*-pAR1219 strain was produced. This was to provide T7 RNA polymerase for the TargeTron^®^ system to function once delivered into the bacterial cell. In the second step, the *E. mallotivora*-pAR1219 strain was transformed again with pΔ*hrpL* to generate putative mutants. Putative mutants were detectable after 48 hours (temperature = 28 °C) of selection on LB agar supplemented with kanamycin (50 μg/mL). To confirm intron insertion into the *hrpL*, PCR was conducted on putative mutant genomic DNA using combinations of gene- and intron-specific primers followed by DNA sequencing. All PCR conditions were as follows: initial pre-denaturation at 94 °C for 2 min followed by 35 cycles of denaturation at 94 °C for 30 s, annealing at 52 °C for 30 s, and extension at 72 °C for 2 min.

### 2.5. Pathogenesis Assay of the Mutant Strain

Four-month-old plants of *Carica papaya* cv. Eksotika I were supplied by the Malaysian Agricultural Research and Development Institute (MARDI) Pontian, Johor. The plants were grown in a greenhouse under standard tropical conditions, where they received 13 h of light a day. Fresh colonies of the wild type and knockout mutant Δ*EmhrpL* strains of *E. mallotivora* were cultured overnight in LB broth (one colony in 50 mL broth) and incubated (with shaking at 150 RPM) at 28 °C until they reached OD_600_ = 1.0. Artificial wounding (by pricking) of the three lowermost papaya leaves was carried out using sterile needles and about five mL of *E. mallotivora* mutant/wild-type culture (resuspended in 1 × phosphate-buffered saline) was sprayed to the plant part ~15 cm from the shoot. Plants sprayed with 1 × phosphate-buffered saline without *E. mallotivora* served as negative controls and all inoculated/sprayed plants were done in triplicates. The scorings were accomplished between 3–30 days post-inoculation (DPI).

Disease severity was scored following the study conducted by Juri et al. [21]. Disease severity scoring was evaluated using a 5-stage scale as follows: 0—symptomless; 1—leaf vein blackening; 2—leaf vein blackening + slight wilting; 3—leaf stalk wilting; 4—stem blackening; 5—plant death.

## 3. Results

### 3.1. Sequence Characterisation of EmhrpL Gene and Its Product, HrpL

**Figure 2:**
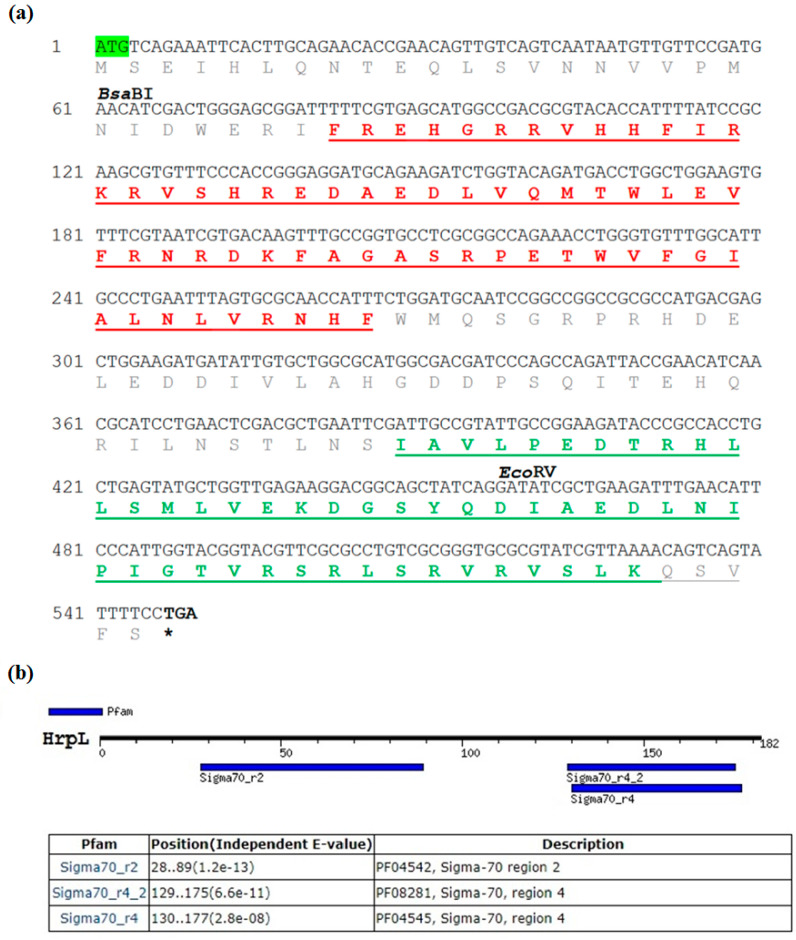

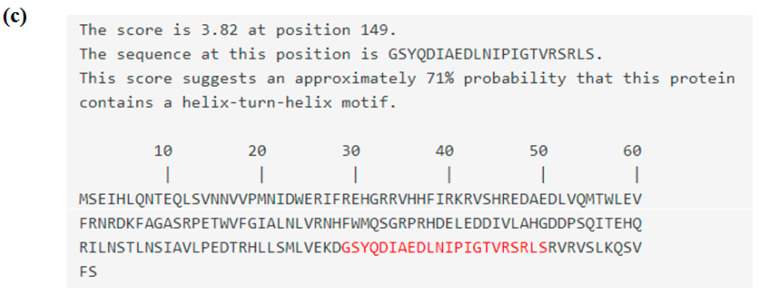
Sequence analysis of *Emhrpl* open reading frame (ORF) and motif finding on its translated protein. (**a**) Nucleotide sequence of *EmhrpL* (MK205448) and its deduced amino acids. The nucleotide sequences for *Bsa*BI and *Eco*RV restriction sites are as indicated and the two conserved σ factor motifs on the amino acids, region R2 (red letters) and region R4 (green letters), are underlined. (**b**) Motif search on GenomeNet detected only two σ regions (or motifs) on *Em*HrpL, indicating the σ protein belongs to Group IV factor while (**c**) NPS@ server identified an HTH motif at position 149 of the amino acid (letters in red) corresponding to σR4 of the *Em*HrpL. (* = termination of translation by the stop codon TGA).

**Figure 3:**
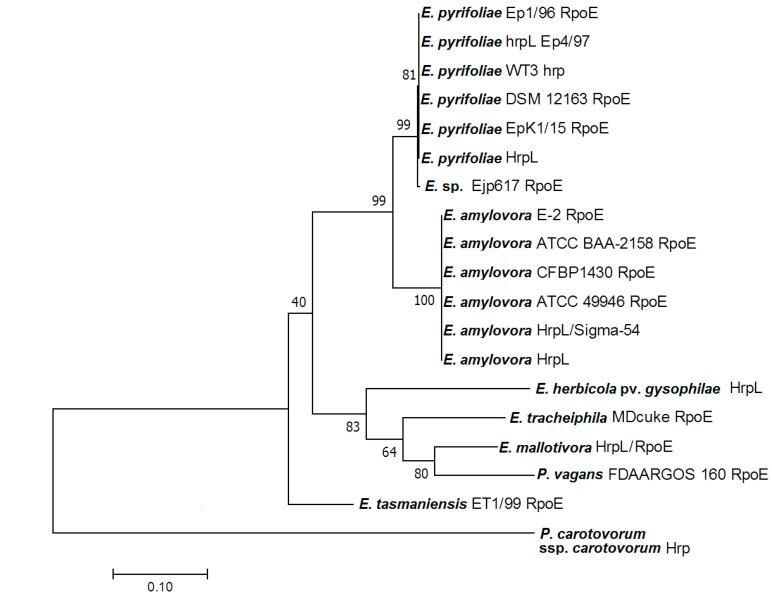
Molecular phylogenetic analysis of *hrpL* from *E. mallotivora* (Accession no. MK205448) and *hrpL*/*rpoE* gene sequences from related taxa by Maximum Likelihood (ML) method. A total of 18 nucleotide sequences were obtained from NCBI to construct the phylogenetic tree of sigma factors, and *Pseudomonas carotovorum* ssp. *carotovorum* served as an outgroup. The evolutionary history was inferred by using the ML method based on the Tamura–Nei model. The tree with the highest log likelihood (−2872.15) is shown.

The full *EmhrpL* CDS was deposited to GenBank under the accession number MK205448. The CDS was 549 bp long and encoded 182 amino acids. Its start codon did not follow the Kozak consensus sequence pattern (ATGG), confirming the *hrpL* belongs to a non-eukaryotic organism (Figure 2a). The calculated molecular weight (MW) of the deduced amino acid was 21,295.03 Da (21.3 kDa), and it had an overall isoelectric point (IP) of 6.19, indicating the gene product belongs to the σ^24^ protein group and is slightly acidic. A sequence search of *EmhrpL* against the NCBI database revealed high sequence similarity of the protein to other alternative σ factors from 18 bacterium species (Table 1) and phylogenetic inference based on those *hrpL*/*rpoE* sequences (Figure 3) indicated *E. mallotivora* was clustered together with *E. tracheiphila* and *Pantoea vagans*—pathogens that cause bacterial diseases in cucurbits and eucalyptus, respectively [22,23].

**Figure 4:**
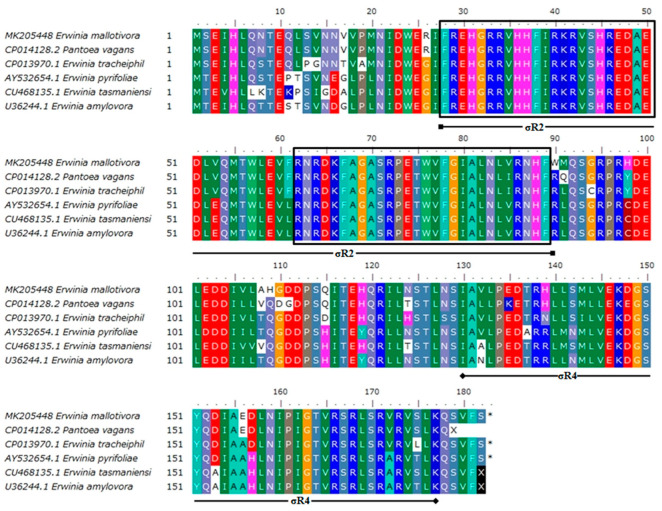
Multiple sequence alignment of deduced amino acids of *E. mallotivora hrpL* (MK205448), *Pantoea vagans rpoE* (CP014128.2), *E. tracheiphila rpoE* (CP013970.1), *E. pyrifoliae hrpL* (AY532654.1), *E. tasmaniensis rpoE* (CU468135.1), and *E. amylovora hrpL* (U36244.1). Boxed are the extremely conserved sequences located on σR2 regions from six different bacterium species.

The GenomeNet motif search tool (Pfam database, https://pfam.xfam.org/) revealed *Em*HrpL harboured two highly conserved σ regions—σR2 and σR4 (Figure 3). Based on the amino acid sequence alignment on six closely related RpoEs/sigmas/HrpLs (Figure 4), σR2 and σR4 of *Em*HrpL were highly conserved and their calculated PIs were 11.11 and 6.78, respectively. Additionally, σR4 was verified using the Pfam database and NPS@ server to have the helix-turn-helix (HTH) DNA binding motif (Figure 2b,c). Protein localisation analysis through PSORTb v3.0.2 predicted *Em*HrpL was a cytoplasmic protein (Table 2) and this result is consistent with the σ factor RpoE from *E. coli* [24,25].

### 3.2. Homology of EmHrpL

**Figure 5:**
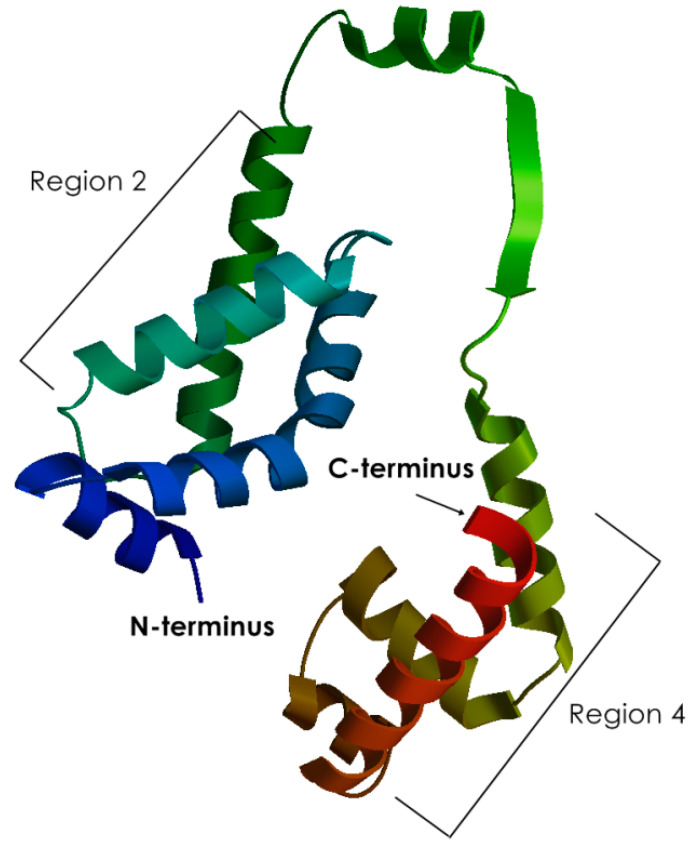
Predicted 3D structure of *E. mallotivora*’s σ factor (100% confidence) based on homology modelling of *E. coli* sigma factor E (RpoE) crystal structure. The region 4 (σR4) structure possesses the helix-turn-helix (HTH) motif as visualised.

To further investigate the protein conformation of *Em*HrpL, a series of in silico tools were employed to generate a 3D structure. The Phyre2 protein fold recognition server had generated a list of homologous proteins based on the submitted amino acid sequence, and the top four models are tabulated in Table 3. Template c1or7A_ (based on the crystal structure of *E. coli* sigma factor E) was selected for *Em*HrpL structure analysis for having the best identity percentage and 100% confidence. The c1or7A_ PDB file was then reconstructed using Phyton Molecular Viewer (PMV 1.5.6, Schrödinger Inc., New York, NY, USA) to visualise the 3D protein structure as in Figure 5. The generated σ 3D model comprised a conserved σ region 2 (σR2) as the N-terminal domain and a conserved σR4 domain as the C-terminal domain. These two regions, or domains, were connected by a σ linker, and this conformation was very similar to the crystal protein structure of *E. coli* RpoE reported by Campbell et al. [26]. In accordance with the previous amino acid sequence analyses, a helix-turn-helix (HTH) motif that mediates σ factor interaction with the −35 element in the promoter region and a cognate anti-σ factor can be visualised from the σR4 domain of the *Em*HrpL 3D structure.

### 3.3. Prediction of a Potential Inhibitor and Its Binding Affinity (Kd) with EmHrpL through PPI In Silico Analysis

**Figure 6:**
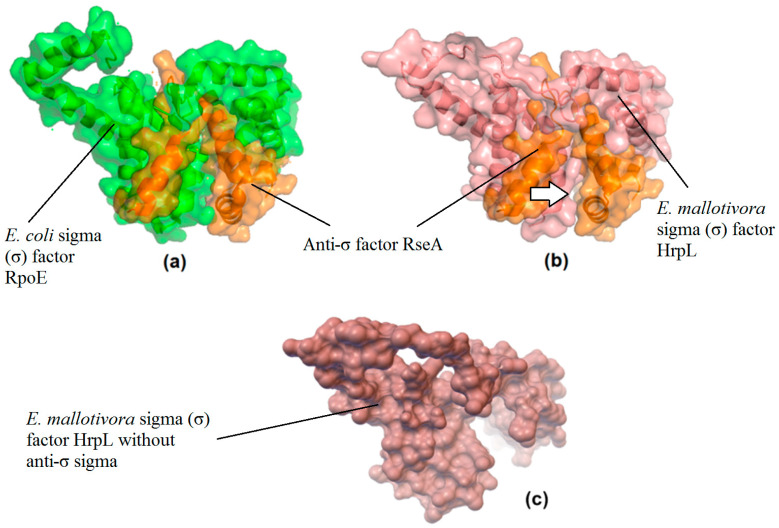
Protein–protein interaction (PPI) study of molecular docking using ZDOCK protein-docking server. Structure as with visible surface at 40% transparency and secondary structures edited in PyMOL. (**a**) *E.coli* RNA polymerase sigma-E factor chain A (green) forming a complex with anti-σ factor RseA chain C (orange), and (**b**) *Em*HrpL (red) bound by anti-σ factor RseA chain C (orange) with a visible gap between the two proteins (white arrow). (**c**) *Em*HrpL without the anti-σ factor RseA viewed using PMV with a visible surface at 0% transparency.

As stated, *E. mallotivora* HrpL protein shared conserved motifs with the *E. coli* σ^E^ (or RpoE); hence, the molecular docking technique was employed to assess the probability of *E. mallotivora* HrpL forming a complex with the *E. coli* anti-σ factor RseA. A molecular docking simulation was conducted using ZDOCK online server version 3.0.2 to investigate the protein–protein interaction (PPI) of the two σ factors with the said inhibitor, and the generated results are revealed in Figure 6. The highest ZDOCK score for the *Em*HrpL:RseA complex was 1312.338, and the 1OR7 (RpoE:RseA) complex was also re-docked to compare its score, which was 3316.534, 40% higher than *Em*HrpL:RseA score. A higher docking score means better interaction affinity when analysed using ZDOCK. The total interface area of the *Em*HrpL:RseA complex was 5223.3 Å^2^ with gap volume 6945.75 Å^3^ forming eight hydrogen bonds and seven salt bridges. On the other hand, the 1OR7 (RpoE:RseA) complex had a total interface area of 5715.3 Å^2^ with gap volume 4522.50 Å^3^ forming 16 hydrogen bonds and seven salt bridges in between. Based on these figures, the interface areas (Å^2^) in both protein complexes were not too different from each other (5715.3 vs. 5223.3 Å^2^), and this indicated that the total contact area between the RseA with HrpL is very similar to that produced by the 1OR7 complex. However, *Em*HrpL:RseA had a much higher gap volume (Å^3^) and formed 50% less hydrogen bonds compared to 1OR7. Based on the ZDOCK score, interface areas, hydrogen bonds, and salt bridges, the binding affinity (Kd) for *Em*HrpL:RseA was estimated to be 0.8 × 10^−10^ M, slightly less than half of what had been determined for the 1OR7 complex, which was 2 × 10^−10^ M [26,27].

According to Janin et al. [28] and Erijman et al. [29], an interface area of ~1500 Å^2^ with at least ten hydrogen bonds has enough enthalpy to generate a dissociation constant (Kd) of up to 10^−14^ M (fM), and the smaller the Kd, the higher the binding affinity between the two substances. Erijman et al. [29] conducted a comprehensive study on different levels of binding affinities on different PPIs based on several types of molecular features and concluded that PPIs can be classified as high (Kd ≤ 10^−9^ M), medium (10^−9^ M < Kd ≤ 10^−6^ M), and low affinity (Kd > 10^−6^ M). As previously mentioned, 1OR7 has been determined to have Kd of 2 × 10^−10^ M, which is in the high-affinity PPI category. Based on the PPI analysis and binding affinity levels produced by Erijman et al. [29], it is concluded that the binding affinity for *Em*HrpL:RseA (0.8 × 10^−10^ M) is estimated to be in the high-affinity PPI, though having Kd slightly lower than that of 1OR7.

### 3.4. Targeted hrpL Disruption in E. mallotivora by Using a Group II Intron (TargeTron^®^) System

**Figure 7:**
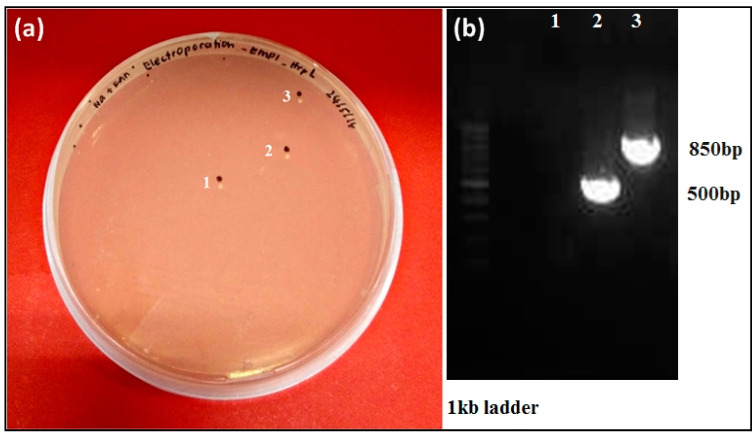
Antibiotic selection of transformed *E. mallotivora* cells and confirmation of *hrpL* disruption in the genome by PCR. (**a**) Selection of putative mutants was carried out on LB agar supplemented with kanamycin (50 μg/mL). (**b**) For PCR validation, *hrpL* gene-specific primers were used to validate the putative mutants obtained from the selection step. The mutated strain (Δ*EmhrpL*) has a larger gene size due to insertion by the intron (lane 3) compared to non-mutant/wild type (lane 2). Lane 1 served as negative control.

In order to further understand how *hrpL* determines *E. mallotivora* pathogenicity in papaya, functional characterisation of this T3SS regulator was conducted through loss-of-function mutagenesis, and a similar gene knockout experiment targeting a different gene was conducted by Juri et al. [20]. After transformation via electroporation, putative mutant colonies were first selected on LB agar containing kanamycin and subsequent PCR had validated the transformed cells containing the intron insertion based on the larger amplicon size (850 vs. 500 bp) (Figure 7b). Colony PCR was also conducted on *E. mallotivora* marker genes (*E. mallotivora* *hrpN*, isochorismate mutase, and *hrpS*) to validate the species authenticity of the Δ*EmhrpL* colonies (data not shown). In this study, the mutant *E. mallotivora* strain (Δ*EmhrpL*) was successfully generated (Figure 7a), though the number of retrieved colonies was very low. The steps were relatively meticulous since the bacteria needed to be transformed with two types of vectors, pAR1219 and pΔ*hrpL* (pACD4K-C).

### 3.5. Mutagenesis Study Revealed Involvement of hrpL in the Pathogenicity of E. mallotivora

To investigate the involvement of *hrpL* in *E. mallotivora* pathogenicity, the Δ*EmhrpL* strain was used to artificially inoculate four-month-old papaya plants and the resulting disease severity was compared against severity produced by wild type *E. mallotivora* (positive control). The progression of dieback disease severity (averaged) on papaya plants caused by Δ*EmhrpL* vs. wild type strain is shown in Table 4 and it was based on the 0–5 papaya dieback disease severity score [21]. Upon infection with the Δ*EmhrpL* strain, the papaya plants manifested zero symptoms during 3–12 DPI. However, the veins of wounded leaves started to blacken (stage 1) on 16–20 DPI, indicating early entry of the mutant *E. mallotivora* through the wounds. However, beyond 25 DPI, the leaf vein blackening of wounded leaves diminished while the leaves slowly turned yellow (data not shown). On 30 DPI, the leaves previously presenting stage 1 symptoms were completely abscised from the stem and only unwounded leaves remained healthy and intact. At this stage, the disease severity score of these plants had reverted to stage 0. Blackening of leaf veins (stage 1) was also observed in papaya plants infected with wild type *E. mallotivora* but it manifested as early as 3 DPI. The progression of disease severity continued from stage 2 through to stage 5 until 20 DPI, where the plants completely succumbed to the disease and were beyond cure. It was observed that the severity and intensity of the symptoms developed by plants sprayed with the mutant *E. mallotivora* strain, Δ*EmhrpL,* was significantly lower than that of plants sprayed with parent*/*wild type *E. mallotivora*.

## 4. Discussion

The T3SS is highly conserved in Gram-negative bacteria and a comparative study on genes from related taxa is very useful in determining common biological features, especially on conserved functions. The importance of *hrpL* and involvement of the T3SS in pathogenicity have been documented in other phytopathogenic bacteria [30,31]. Wei and Beer [13] reported the *hrpL* of *E. amylovora* is involved in the *hrp* signal transduction cascade during plant–pathogen interaction in the fire blight disease of rosaceous plants; thus, the *hrpL* from *E. mallotivora* should have a similar role in causing infection in papaya due to the conserved motifs identified from the nucleotide and amino acid sequences. In this study, the sequence analyses indicated *E. mallotivora* HrpL (*Em*HrpL) belongs to an extracytoplasmic function (ECF) protein family whose members are classified based on their generally smaller size and the presence of only two σ regions (σR2 and σR4) [32,33]. The ECF proteins, as the name indicates, regulate gene expressions pertaining to stresses and pathogenesis upon detection of stimuli that come from the exterior of the cell cytoplasm [2,13,34,35]. The two highly conserved σR2 and σR4 regions that specified *Em*HrpL belong to a larger, primary sigma factor σ^70^ family and the absence of σR1.1 has confirmed that the protein belongs to Group IV σ factor [36,37]. According to the literature, the two sigma regions (σR2 and σR4) are crucial for RNA polymerase DNA-specific recognition during transcription initiation, where the σR2 is responsible for -10 promoter recognition while σR4 is involved in -35 promoter binding during RNA polymerase interaction with the gene promoters [9,27,36,38,39]. In addition, σR2 and σR4 are the sites pairing up with a cognate anti-σ during inhibition, and this feature has gained the main interest in this study. According to Lonetto et al. [36], these two sigma R2 and R4 regions could be further divided into several subregions but these are not discussed in this article.

Jovanovic et al. [40] reported that regulatory factor HrpV is able to impose negative control on *hrpL* gene expression in *P. syringae—*a Gram-negative bacterium related to *Erwinia* spp.—though not through a direct interaction. A transcriptomics study reported by Juri et al. [20] managed to identify a set of pathogenesis-related genes during the early hours of *E. mallotivora* infection and *hrpV* was one of the differentially expressed genes (DEGs), but the correlation of this gene with other T3SS elements in *E. mallotivora* was not discussed. It was then decided to attend to a different element that has a more direct interaction with HrpL through the σR2 and σR4 sites. There has not been any report on HrpL σ factor from *Erwinia* being directly regulated, or inhibited, by a cognate anti-σ—a type of negative regulator [41]. However, in *E. coli*, its σ factor RpoE (homologous to HrpL) remains inactive and bound to a cognate anti-σ factor, RseA, inside the cytoplasm until an extracytoplasmic stimulus triggers the release of RpoE [26]. It was also shown by Campbell et al. [26] and Tam et al. [27] that *E. coli* σ^E^ (RpoE) was strongly inhibited by the anti-σ factor RseA with binding affinity (Kd) of 0.2 nM (2×10^−10^ M) through the formation of σ^E^:RseA (or RpoE:RseA) complex (PDB ID: 1OR7). Generally, σ factors are co-transcribed with a cognate negative regulator and remain inactive by forming the σ: inhibitor complex. The σ factors will only be released from the complex to become ECF proteins once they receive a stimulus from the environment [6,35]. Since the anti-σ factor RseA is a small protein that originally serves to inactivate RpoE in *E. coli*, its potential as an inhibitory molecule to suppress T3SS in *E. mallotivora* is very appealing to research. Our previous studies on transcriptomics, proteomics, and the draft genome were not able to identify the cognate anti-σ factor for *Em*HrpL [18,19,20]; hence, a bioinformatics simulation had to be run to predict the bipartite interaction of *E. coli* RseA with the σR2 and σR4 regions on *E. mallotivora* HrpL. As produced by the PPI study, it was simulated that the binding affinity of RseA to *Em*HrpL is comparable to that of native complex RpoE:RseA; therefore, the anti-σ factor RseA has the potential to be used as an inhibitor to suppress the T3SS pathway in *E. mallotivora* and could be incorporated for disease control approaches. In addition to this, Boldrin et al. [42] concretely proved the inhibitory interaction of Rv1222 (RseA) on the expression of σ^E^-dependent genes in *Mycobacterium tuberculosis,* and this aligns with our result on the docking simulation.

In the final part of the experiment, further investigation was performed for the involvement of *EmhrpL* in causing papaya dieback disease through a loss-of-function mutagenesis study using a type II intron system (TargeTron^®^). As mentioned, it was observed Δ*EmhrpL* exhibited ‘weakened’ pathogenicity based on reduced symptoms in infected plants (stage 1) instead of complete nullification. Juri et al. [19] and Abu-Bakar et al. [20] reported T3SS was not the only means of pathogenesis in *E. mallotivora*; hence, hypersensitive response (HR) could still be observed, though it occurred much later and was less aggressive compared to the control set. The possibility of the type II intron insertion in Δ*EmhrpL* to be lost when the symptom appeared on 16 DPI is ruled out since this system has been tested to be stable [43]. A mutagenesis study was done on *M. tuberculosis* by disrupting the σ^E^ gene, and it was discovered that the mutant *M. tuberculosis* had a reduced lethality in mice [44], a finding that resonates with our current study. Thus, it is concluded that *E. mallotivora* highly depends on HrpL to cause disease in its host plant, *C. papaya*.

So far, *E. mallotivora* has been reported to infect *Mallotus japonicus* and Malaysian *C. papaya* cultivars [17,45], while other possible hosts are unknown. Based on the present study, it is evident that *hrpL* plays a pivotal role in *E. mallotivora* pathogenesis. To date, there is no published study on the HrpL/RpoE interaction with an anti-σ in plant pathogens and our work provides the preliminary evidence of such interaction in *E. mallotivora.*

## 5. Conclusions

In this study, the phylogenetic relationship of *EmhrpL* with other sigma factors across many Gram-negative pathogens has been inferred and they harbour much conserved motifs, signifying a unified function in the pathogen-related pathway. In silico structural properties of *Em*HrpL protein have been identified, and its possible interaction with an anti-σ has been simulated through molecular docking analysis. Even though the cognate, or native, *Em*HrpL anti-σ is yet to be determined, there is a conviction that *Em*HrpL is indeed regulated by one. *Erwinia mallotivora hrpL*’s important role in causing papaya dieback disease has ultimately been confirmed in a mutagenesis study, and this information is consistent with results produced from other studies. The outcomes of this project, especially on the role of an anti-σ, have improved our understanding of a regulatory element related to pathogenesis in *E. mallotivora*.

## Figures and Tables

**Table 1:** Summary of BLASTn results based on the *EmhrpL* sequence (Accession no. MK205448).

Description	Max Score	Total Score	Query Cover	E Value	Identity	Accession
*Pantoe vagans* FDAARGOS 160	598	598	97%	8 × 10^−167^	85%	CP014128.2
*Erwinia tracheiphila* MDcuke	576	576	100%	3 × 10^−160^	83%	CP013970.1
*Erwinia* sp. Ejp617,	519	519	99%	6 × 10^−143^	81%	CP002124.1
*E. pyrifoliae* EpK1/15	510	510	99%	3 × 10^−140^	81%	CP023567.1
*E. pyrifoliae* DSM 12163	510	510	99%	3 × 10^−140^	81%	FN392235.1
*E. pyrifoliae* WT3	510	510	99%	3 × 10^−140^	81%	DQ180962.2
*E. pyrifoliae* Ep1/96	510	510	99%	3 × 10^−140^	81%	FP236842.1
*E. pyrifoliae*	510	510	99%	3 × 10^−140^	81%	AY532654.1
*E. pyrifoliae* Ep4/97	510	510	99%	3 × 10^−140^	81%	AJ438881.1
*E. tasmaniensis* ET1/99	488	488	99%	1 × 10^−133^	80%	CU468135.1
*E. amylovora* E-2	465	465	99%	1 × 10^−126^	79%	CP024970.1
*E. amylovora* ATCC BAA-2158	465	465	99%	1 × 10^−126^	79%	FR719186.1
*E. amylovora* CFBP1430	465	465	99%	1 × 10^−126^	79%	FN434113.1
*E. amylovora* ATCC 49946	465	465	99%	1 × 10^−126^	79%	FN666575.1
*E. amylovora*	465	465	99%	1 × 10^−126^	79%	AF083877.1
*E. amylovora*	465	465	99%	1 × 10^−126^	79%	U36244.1
*E. herbicola* pv*. gypsophilae*	445	445	100%	1 × 10^−120^	78%	AF272053.1
*Pseudomonas carotovorum* ssp. *carotovorum*	109	109	73%	1 × 10^−19^	67%	EU420066.1

**Table 2:** Tabulated result on prediction of subcellular localisation of HrpL using PSORTb v3.0.2 Bacterial Localisation Prediction Tool.

Seq ID: MK205448.1		
*Erwinia mallotivora* strain BT-MARDI RNA Polymerase Sigma Factor RpoE/hrpL		
Analysis Report		
Analytical Modules	Prediction	Details
CMSVM	Unknown	-
CytoSVM	Cytoplasmic	-
SCL-BLAST	Cytoplasmic	Matched 16130498: RNA polymerase, sigma 24 (sigma E) factor [*Escherichia coli* K12]
SCL-BLAST	Unknown	-
Signal	Unknown	-
**Localisation**	**Scores**	
Cytoplasmic	9.97	
Cytoplasmic Membrane	0.01	
Periplasmic	0.01	
Outer membrane	0.00	
Extracellular	0.00	
**Final Prediction**		
Cytoplasmic	9.97	

**Table 3:** Homology modelling of *EmhrpL* from top four models (100% confidence). Primary amino acids sequence of *EmhrpL* was submitted to Phyre2 and targeted with sigma factors available on PDB.

Template	Alignment Coverage	Confidence	% Identity	Template Formation
c1or7A_	8–181 (95%)	100	23	**PDB header**: transcription **Chain**: A: **PDB Molecule**: RNA polymerase sigmaE factor; **PDB Title**: crystal structure of *Escherichia coli* sigmaE with the cytoplasmic domain of its anti-sigma RseA
c4cxfA_	7–182 (96%)	100	20	**PDB header**: transcription **Chain**: A: **PDB Molecule**: RNA polymerase sigma factor CnrH; **PDB Title**: structure of CnrH in complex with the cytosolic domain of CnrY
c5wurB_	7–181 (95%)	100	23	**PDB header**: metal-binding protein **Chain**: B: **PDB Molecule**: ECF RNA polymerase sigma factor SigW; PDB Title: crystal structure of SigW in complex with its anti-sigma RsiW, an oxidised form
c2q1zA_	5–180 (96%)	100	18	**PDB header**: transcription **Chain**: A: **PDB Molecule**: RpoE, ECF SigE; **PDB Title**: crystal structure of *Rhodobacter sphaeroides* SigE in complex with the anti-sigma ChrR

**Table 4:** Summarised dieback disease severity scoring of papaya plants (in triplicates) after being sprayed/inoculated with wild type strain, Δ*EmhrpL*, and negative control solution (Stage 0—symptomless; Stage 1—leaf vein blackening; Stage 2—leaf vein blackening + slight wilting; Stage 3—leaf stalk wilting; Stage 4—stem blackening; Stage 5—plant death).

Culture/Suspension	Averaged Scoring of Infection (Disease Severity)						
	Day3	Day6	Day9	Day12	Day16	Day20	Day30
Wild type *E. mallotivora*	1	2	2	3	4	5	5
Knockout mutant, Δ*EmhrpL*	0	0	0	0	1	1	0
1× phosphate-buffered saline (negative control)	0	0	0	0	0	0	0
Strain of *Erwinia mallotivora*	Disease progression Day 3 (D3) until Day 30 (D30) post inoculation with respective *E. mallotivora* strain						
Wild Type (Control)	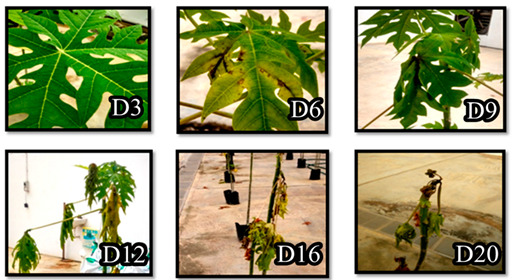						
Knockout Mutant (Δ*EmhrpL*)